# The Effect of Repetitive Transcranial Magnetic Stimulation (rTMS) on Cognition in Patients With Traumatic Brain Injury: A Protocol for a Randomized Controlled Trial

**DOI:** 10.3389/fneur.2022.832818

**Published:** 2022-03-30

**Authors:** Han Zhang, Yu Zhao, Yun Qu, Yunyun Huang, Zhu Chen, Hong Lan, Yi Peng, Hongying Ren

**Affiliations:** ^1^Department of Rehabilitation Medicine, West China Hospital of Sichuan University, Chengdu, China; ^2^Department of Rehabilitation Medicine, Second Clinical Medical College of North Sichuan Medical College, Nanchong Central Hospital, Nanchong, China; ^3^College of Rehabilitation Medicine, West China Hospital of Sichuan University, Chengdu, China; ^4^Sichuan Provincial Key Laboratory of Rehabilitation Medicine, Sichuan University, Chengdu, China

**Keywords:** traumatic brain injury, repetitive transcranial magnetic stimulation, memory, executive function, cognitive impairment

## Abstract

Cognitive impairment, defined as a decline in memory and executive function, is one of the most severe complications of traumatic brain injury (TBI). Patients with TBI are often unable to return to work due to cognitive impairment and their overall quality of life is reduced. TBI can bring a serious economic burden to patient's families and to society. Reported findings on the efficacy of repetitive transcranial magnetic stimulation (rTMS) in improving cognitive impairment following TBI are inconsistent. The purpose of the proposed study is to investigate whether rTMS can improve memory and executive function in patients with TBI. Herein, we propose a prospective randomized placebo-controlled (rTMS, sham rTMS, cognitive training), parallel-group, single-center trial. 36 participants with a TBI occurring at least 6 months prior will be recruited from an inpatient rehabilitation center. Participants will be randomly assigned to the real rTMS, sham rTMS, or cognitive training groups with a ratio of 1:1:1. A 20-session transcranial magnetic stimulation protocol will be applied to the left and right dorsolateral prefrontal cortices (DLPFC) at frequencies of 10 Hz and 1 Hz, respectively. Neuropsychological assessments will be performed at four time points: baseline, after the 10th rTMS session, after the 20th rTMS session, and 30 days post-intervention. The primary outcome is change in executive function assessed using the Shape Trail Test (STT). The secondary outcome measures are measures from neuropsychological tests: the Hopkins Verbal Learning Test (HVLT), the Brief Visuospatial Memory Test (BVMT), the Digit Span Test (DST). We report on positive preliminary results in terms of improving memory and executive function as well as beneficial changes in brain connectivity among TBI patients undergoing rTMS and hypothesize that we will obtain similar results in the proposed study.

## Introduction

The Centers for Disease Control and Prevention (CDC) defines a traumatic brain injury (TBI) as a mechanical injury to the brain caused by a collision, blow, or bump (1). TBI is a major health problem worldwide owing to the complex complications and sequelae associated with this condition. TBI affects more than 50 million people worldwide each year, including approximately 1.7 million people in the United States alone (2, 3). Although effective treatment in the acute phase has greatly improved survival among TBI patients, an estimated 5–7 million TBI patients in Europe and the United States suffer from multiple complications.

Cognitive impairment is the main clinical symptom of TBI. Cognitive impairment following TBI is often characterized by attention deficits, memory loss, and decreased executive ability. TBI can initiate a long-term neurodegenerative process that leads to pathological features similar to those of Alzheimer's disease (AD) (4–6). More than 50% of TBI patients experienced an impaired state of consciousness (i.e., a coma or a vegetative state), which has reported to persist in the form of long-term cognitive impairment (7). It is well known that deficits in executive function and memory are commonly detected within TBI, and these deficits are more difficult to treat as compared with deficits associated with other cognitive disorders as a result of the vulnerability of the affected brain regions (such as the frontal and temporal lobes). These sequalae negatively affect patients' quality of life as well as their rehabilitation process (8). Numerous studies have demonstrated that cognitive training can improve multiple cognitive domains inTBI involving reasoning, goal management, attention, as well as reorganized modular networks (9–11).

Recently, repetitive transcranial magnetic stimulation (rTMS), a neuromodulatory tool, can induce neural activity and efficiently improve cognitive function (12–15). A large number of existing studies confirm that repetitive transcranial magnetic stimulation (rTMS) of the dorsolateral prefrontal cortices (DLPFC) may strengthen the connections within the brain network, which may increase the strength of the interconnections between the cortex and hippocampus, resulting in cognitive enhancement (16, 17). High frequency (≥ 5) rTMS (HF-rTMS) is the most widely used technique to improve cognitive function in clinical trials as well as in healthy individuals (18). In a review of cognitive enhancemen using rTMS, 10 Hz was found to be the frequency used in most studies (19). Most studies found that HF-rTMS on left DLPFC improved memory and processing skills in healthy, depressed, posttraumatic stress disorder (PTSD), schizophrenic, AD, and mild cognitive impairment (MCI) populations. However, some showed no significant effect. In another review of the efficacy of LF-rTMS on cognition, the findings of 1 Hz rTMS on the right DLPFC on improving cognitive function in mood disorders, psychosis, stroke and other organic disorders were also inconsistent (20).

A Systematic Review Also Reported That RTMS Enhances Neuroprotection and Recovery Following TBI in Animals (21). However, There Have Been Fewer Studies Investigating the Effects of RTMS on Cognitive Function in Humans Presenting With TBI, and Most Investigations to Date Have Been Clinical Case Reports or Case Series. To the Best of Our Knowledge, Only two RCTs Have Been Reported to Dates Neville et al. Reported That Stimulating the Left DLPFC With 10 Hz RTMS Did not Improve Cognitive Function in Subjects, While Lee and Kim Stimulated the Right DLPFC With 1 Hz RTMS, Which Improved Cognitive Function in Subjects (22, 23). They Reported the Safety and Efficacy of Implementing RTMS on Bilateral DLPFC and may be an Alternative Type of Intervention to Improve Cognitive Function. Also, Some Studies Suggested That at Least 4 Weeks of RTMS Intervention may be Necessary to Improve Cognitive Function.

Therefore, the proposed study aims to examine HF and LF, bilateral rTMS for the cognitive dysfunction in patients with TBI. In addition, magnetic resonance imaging (MRI) will be utilized to explore the association between brain structure and functional connectivity, as well as the cognitive effects of rTMS. The preliminary hypotheses of our study are (1) 4 weeks of bilateral high- and low-frequency rTMS is more effective in improving cognitive function in patients with TBI than sham stimulation and cognitive training and (2) structure MRI markers and functional connectivity pre- and post-intervention will be observed.

## Materials and Methods

### Study Design

**Figure 1:**
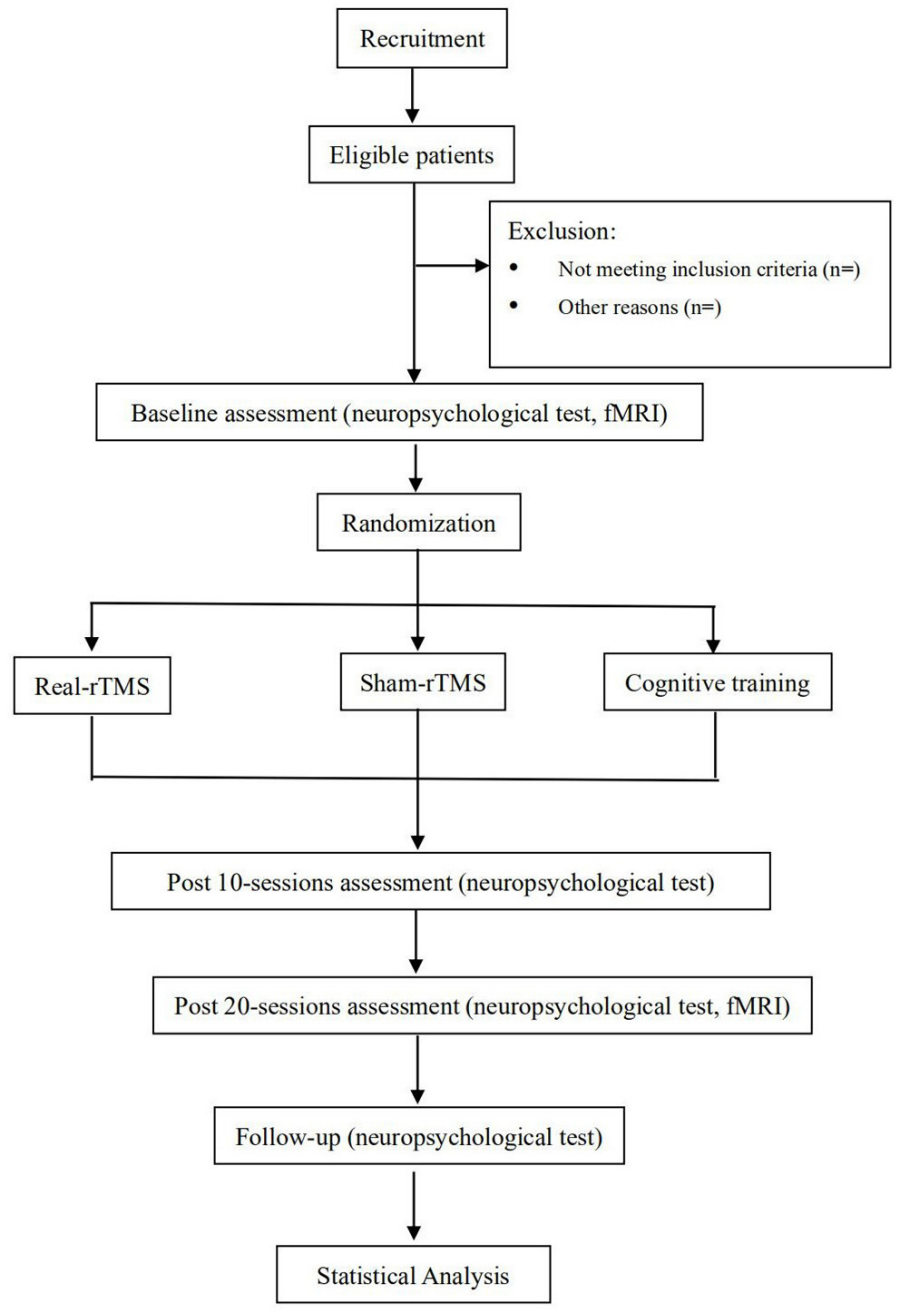
Flowchart of the study design. rTMS, Repetitive Transcranial Magnetic Stimulation; fMRI, functional Magnetic Resonance Imaging.

The study is a prospective randomized placebo controlled, parallel-group trial that will be conducted at the Affiliated Hospital and the Second Clinical Medical College of North Sichuan Medical University. The protocol has been registered with the China Clinical Trial Registry (item number: ChiCTR2100050753). Our study will show improvement in cognitive function in patients with TBI following rTMS bilateral DLPFC intervention. The findings may provide a rationale for an approach of rTMS intervention for cognitive function in patients with TBI. Thirty-six patients with TBI will be enrolled in the trial. The physicians will provide patients with detailed information about the trial in verbal and written form before the trial begins.All patients will voluntarily sign an informed consent form. All patients will be screened according to eligibility criteria. 36 subjects will be randomized in a 1:1:1 ratio into the real-rTMS group, the Cognitive training group and the sham-rTMS group. All subjects will be required to perform neuropsychological assessments at before intervention, post-10 sessions, post-20 sessions, and 30 days after the intervention. MRI will be performed at pre-intervention and post-20 sessions. All patients will receive intervention for 4 weeks (30 minutes per day, 5 days per week). The diagram and schedule for the study are shown in Figure 1 and Table 1.

**Table 1:** Schedule of enrollment, assessment, and interventions.

			**Screening**	**Randomization**	**Treatment**				**Follow up**
					**10 sessions**		**20 sessions**		
	**Timepoint**		**Within two weeks**	**Day 0**	**Week 1**	**Week 2**	**Week 3**	**Week 4**	**Post-30 day**
				**T1**	**T2**		**T3**		**T4**
Enrollment
	Informed consent		√						
	Demographic information		√						
	Medical history		√						
	Eligibility assessment		√						
	Radom allocation			√					
Interventions	Real rTMS				√	√	√	√	
	Sham rTMS				√	√	√	√	
	Cognitive training				√	√	√	√	
Assessment	Screening outcome
	Awareness status	RLAS	√					
	Intellectual functioning	MoCA	√					
	Primary outcome			√	√		√		√
	Executive function	STT, DST					
	Secondary outcomes			√	√		√		√
	Memory	HVLT, BVMT					
	Brain connectivity	fMRI			√			√	

*RLAS, Rancho Los Amigos Scale; DST, Digit Span Test; MoCA, The Montreal Cognitive Assessment; rTMS, repetitive transcranial magnetic stimulation; STT, The Shape Trail Test; HVLT, Hopkins Verbal Learning Test; BVMT, Brief Visuospatial Memory Test; fMRI, functional Magnetic Resonance Imaging*.

### Ethical Approval and Consent to Participate

The study has been approved by the Ethics Committee of Nanchong Central Hospital. All participants will provide written informed consent forms at the time of recruitment. Moreover, all study procedures will be conducted in accordance with the principles of the Declaration of Helsinki in its later amendments. Written informed consent will be provided by the participants or their legal guardians.

### Sample and Recruitment

TBI patients will be recruited through inpatient units at the Affiliated Hospital and the Second Clinical Medical College of North Sichuan Medical University. Recruited participants will be required to present with a clear diagnosis of TBI according to U.S. Centers for Disease Control and Prevention (CDC) guidelines based on clinical and imaging findings. The informed consent process will be carried out by the Principal Investigator. Once participants agree to sign the informed consent form, they will be screened to confirm that they are eligible for study participation according to the following inclusion criteria.

### Inclusion Criteria

(a) Adult patients older than 18 and younger than 60 years.
(b) Rancho Los Amigos Scale of Cognitive Functioning (RLAS) score categorizations >VI.
(c) Montreal Cognitive Assessment (MoCA) scores <26.
(d) Diagnosis of TBI occurring ≥ 6 months prior.
(e) patient is right-handed.

### Exclusion Criteria

(a) History of mental or psychological illness.
(b) An implanted device or metal in the brain or body.
(c) Skull defect.
(d) History of epilepsy.
(e) Cardiac pacemaker or cochlear implant.

### Sample Size Estimation

Sample size calculations were based on non-inferiority tests, as the purpose of this study was to show that the rTMS intervention was not inferior to cognitive training. According to studies showing that the effect size of working memory training on MCI is 0.74 and executive function is 0.575, we calculated the sample size using PASS 15 (effect size is 0.7, α is 0.05, power is 0.9). sample sizes of *n* = 10, *n* = 10, and *n* = 10 are obtained for the three groups. Moreover, two additional patients will be added to each group to compensate for possible dropouts. A total of 12 patients will be allocated to each group.

### Randomization

A computer-generated list of random numbers will be placed in an order-numbered, non-transparent, sealed envelope, and participants will be randomly assigned to real rTMS, sham rTMS, and cognitive training groups in a 1:1:1.

### Blinding

It is impossible for a supervised intervention study to be double-blind, only the investigators who performed the assessment and MRI scanning are blinded to group assignment. In further, participants are blinded to the specific hypothesis of the study. To reach proper blinding, we have selected real and sham coils that are similar in shape, size, color, and weight, and that emit extremely similar sounds. The participants, their relatives and the evaluating neuropsychologist will be blinded to group allocation and the timing of the intervention will be set at different times in order to minimize the loss of blinding. All assessments will be conducted through a blinded database deliciated through “A” or “B” labels. Participants will be grouped by investigators not involved in the intervention. Moreover, participants will be unblinded participants when any clinical situation associated with adverse events or patient withdrawal occurs.

### Interventions

Subjects will be screened according to their demographic information and medical history. Upon confirmation of participant eligibility following baseline assessments, participants will be randomly allocated to sham rTMS groups, real rTMS groups, and cognitive training groups in a 1:1:1 ratio. A total of four neuropsychological assessments will be performed: at baseline (T1), after the 10th rTMS session (T2), after the 20th rTMS session (T3), and 30 days following rTMS (T4).

### rTMS

rTMS with a focal figure-of-8-shaped coil (i.e., active and sham coils) will be performed among all participants. The sham coil has the same size and shape as the active coil. However, it does not create any significant magnetic field.

The coils of the transcranial magnetic stimulator will be placed on the patient's left and right dorsolateral prefrontal cortices (DLPFC) based on the F3 and F4 positioning specified in the International 10–20 system for conducting electroencephalography (EEG) (24). The stimulation intensity will be set at a 100% threshold by measuring the threshold of the hallux abductor muscle. rTMS will be delivered to the left DLPFC at a frequency of 10 Hz (5 s durations, 25 s pauses) for a total of 2,000 pulses (50 pulses per train, 40 trains). rTMS will likewise be delivered to the right DLPFC at a frequency of 1 Hz (5 s durations, 25 s pauses) for a total of 2,000 pulses (40 pulses per train, 50 trains). There will be a total of 20 intervention sessions.

#### Cognitive Training

Subjects will be given computer-assisted cognitive training. The computer system is used for cognitive function training, and patients are guided by professionally trained therapists. The task areas include: (a) visuospatial training; (b) executive function and problem solving; (c) attention training; (d) memory training; and (e) calculation training. There are over 20 different therapeutic tasks for training, and the difficulty of the tasks in each area increases automatically as the participant successfully performs the simpler tasks. The cognitive training schedule was 30 min/session, 1 session/day, 5 days/week, with a treatment period of 4 weeks.

### Dropout Criteria

The intervention will be discontinued if the subject meets one or more of the following criteria (a) Subjects who are enrolled incorrectly; (b) Subjects have poor compliance and do not follow treatment as required, as when subjects fail to cooperate with investigators, or when subjects are unable to remain quiet and immobile, or when subjects do not come to treatment on time; (c) Inadequate medical record that interferes with efficacy or safety evaluation; (d) Subjects drop out voluntarily; (e) The subject develops adverse events (including seizures, pain, irritability, etc.); (f) There is severe progression of the subject's condition or some comorbidities, complications, or specific physiological changes; and (g) The subject is considered by the investigator to be unsuitable for further participation in this study.

### Screening Assessment

#### Rancho Los Amigos Scale of Cognitive Functioning (RLAS)

At present, the severity of cognitive impairment following TBI is considered a spectrum that ranges from I to X based on Rancho Los Amigos Scale of Cognitive Functioning (RLAS) scoring. RLAS describes ten states of cognitive and behavioral functioning that patients with brain injury typically progress through. Higher values indicate increased functioning (25).

#### The Chinese Version of Montreal Cognitive Assessment (MoCA)

This assessment scale is comprised of domains measuring attention and concentration, executive functioning, memory, language, visuospatial ability, thinking, calculation, and orientation. The scale consists of eight cognitive domains for which subjects are required to 30 questions. Each correct answer is given 1 score, while incorrect answers or no answers are given 0 score. The cut-off value for the MoCA in the Chinese population is ≥26 (26).

### Outcome Measures

The primary outcome of the proposed study is current executive functioning as measured by the Shape Trail Test (STT), which was revised for the Chinese-speaking population based on the Trail Making Test (TMT). The secondary outcome measures are neuropsychological test findings based on the Hopkins Verbal Learning Test (HVLT), the Brief Visuospatial Memory Test (BVMT), and the Digit Span Test (DST), as well as evaluations of the safety and tolerability of rTMS in patients with TBI.

### Neuropsychological Tests

#### STT

The STT comprises a series of Arabic numerals combined based on Chinese cultural competency and consisting of two parts (27). Part A consists of 25 digits. The participants are asked to connect the numbers as instructed prior to the test. Part B likewise contains 25 digits. However, each digit appears twice (in a circle and in a square). The digits need to be alternately connected by the participants as pre-instructed. The time required for the subject to complete the task will be recorded, less time suggesting better executive function. The test shows an acceptable level with respect to area under the curve (AUC = 0.835), sensitivity (84.6%), and specificity (66.7%) values among elderly people (> 65 years) with an education level <12 years (26).

#### HVLT

This tool assesses memory impairment during vocabulary learning, including evaluations of immediate and delayed memory (28, 29). The test consists of 12 words (three categories, four words per category). Once the words are removed following each display, the participants are immediately asked to recall as many words as they can. There is no requirement for order of recall and there is no time limit for this test. The number of correct words is recorded to calculate the total score. The procedure will be repeated three times for a total score range of 0–36. The test is structured such that a higher score delineates a better memory.

#### BVMT

This test is used to measure visuospatial memory (30). In this test, the examiner presents the participant with a scale consisting of six geometric figures for a total of 10 s each. The participant is then asked to draw as many figures as possible in the correct order. The test is repeated three times. Each figure drawn by the subject was scored according to the criteria provided in the test booklet. Two points are awarded for each figure if it is accurately reproduced and correctly located. Those that are properly placed or drawn correctly receive 1 point. Those with blank answer sheets or no identifiable picture found receive 0 points. The possible scores for a single test range from 0 to 12. The total score is the sum of the scores from the three trials. The test is structured such that a higher score delineates a better memory.

#### DST

The digit span test consists of a forward number test and a backward number test (31). In these two subtests, the examiner reads out a series of numbers to the participant. Participants are asked to repeat the numbers in the same or the opposite order. The digits range from 1 to 9, and the series length is from 2 to 9, for each series length with two trials (e.g., 4–6, 7–5, 2–4–7, 9–6–3, etc.). The test was stopped when the subject missed two trials of a series length. Memory span were scored as the number of items in the longest series recalled forward and backward.

#### Functional Magnetic Resonance Imaging (fMRI) Data Acquisition

Imaging for all participants will be acquired using a Siemens 3.0 T magnetic resonance scanner (Siemens MAGENETOM Trio; Siemens Healthcare, Malvern, PA, USA). The main sequences of the MRI scan and their parameters are as follows. For 3D–T1 weighted imaging, the spoiled Gradient-echo sequence will be selected with the following scan parameters: TR = l,900 ms, TE = 2.48 ms, field of view (FOV) = 250 × 250 mm, layer thickness = 1 mm, layer spacing = 0 mm, matrix = 256 × 256, and flip angle (FA) = 900, with a total of 176 layers. For resting-state fMRI, an echo-planar imaging sequence (EPI) sequence will be selected with the following scan parameters: TR = 2,000 ms, TE = 25 ms, and field of view (FOV) = 240 × 240. The parameters of the FLAIR (fluid-attenuated inversion recovery) sequence are as follows: TR = 8,500 ms, TE = 94 ms, field of view (FOV) = 250 × 250 mm, layer thickness = 0 mm, matrix = 64 × 64, flip angle (FA) = 900, number of layers = 36, total time points acquired = 240 [250 × 250 mm, layer thickness = 5 mm, number of layers = 20, and voxel size = 1.3 × 0.9 × 5 mm^3^]). The scanning time required for each participant is approximately 30 min.

#### Rs-fMRI Data Analysis

Resting-state fMRI images will be preprocessed and regional homogeneity (ReHo) analyses will be performed using DPARSF (Data Processing Assistant for Resting-State fMRI), REST (Resting-State fMRI Data Analysis Toolkit), and SPM8 (Statistical Parametric Mapping) software. Matrix Lab (Matlab2012b; MathWorks, Natick, MA, USA) has been chosen as the platform for data processing. In consideration the time required for participants to adapt to the scanning environment, we will remove the first ten time points from each subject's blood oxygen level dependent (BOLD) sequence; the remaining 230 time points will be subjected to time series corrections, head movement corrections, and head movement parameter tests. If the participants' head movement angle is **>** 2.00 at any angle of x, y, or z or if the maximum translational head movement is **>** 2.0 mm, the participant's data will be rejected. Next, the data space for each participant will be normalized to the Montreal Neurological Institute (MNI) standard space with resampled voxels of 3 × 3 × 3 mm. Six head movement parameters, mean white matter signals, mean whole brain signals, and mean cerebrospinal fluid signals will be removed as covariates and then subjected to de-linearization drift and filtering processing (0.01–0.08 Hz) to eliminate high frequency noise, such as from respiration and heartbeats. Based on the above processed data (i.e., for the ReHo analysis), the ReHo value of each voxel, termed Kendall's coefficient, will be obtained by calculating the consistency of the time series of each voxel and its surrounding adjacent 26 voxels. The ReHo value for each voxel of the whole brain comprises a complete ReHo map. For normalization, the ReHo map of each individual is divided by the average ReHo value of the whole brain. The final obtained image will then be smoothed with full width at half maximum (FWHM) = 4 × 4 × 4 mm. Post-processing of ALFF will likewise be analyzed using REST software. After combining the six cephalomotor parameters, the mean white matter signals, the mean whole brain signals, and the mean cerebrospinal fluid signals will be de-linearized and filtered (0.01–0.08 Hz) before being removed as covariates. After that, the filtered time series will be transformed to the frequency domain range via Fourier transform and a power spectrum at 0.01–0.08 Hz will be calculated. The square root of the power spectrum will be designated as the ALFF value. Each voxel-split ALFF value is divided by the mean value of all voxel ALFFs in order to obtain normalized mean ALFF values. Finally, all covariates will be removed. Functional connectivity analyses will be performed using ALFF with the brain regions of intergroup differences obtained by the ReHo method as seed points in order to assess whole-brain functional connectivity via gray matter templates.

### Adverse Events

A safety questionnaire will be administered to all participants prior to the administration of the first rTMS in order to reduce the occurrence of risk. Possible symptoms, including seizures, tingling, burning sensations, headaches, pain at the electrode site, drowsiness, transient hearing changes, and transient acute hypomania induction (32), will be recorded at the end of each session. Although no adverse effects were found in the published trials on cognitive training reviewed by the committee, and there is little evidence of negative effects of cognitive training. However, any adverse events and negative reactions will be recorded. Descriptive statistics will be performed for all adverse events.

#### Potential Pitfalls and Contingency Plans

Adverse events attributable to rTMS may include seizures, headache, transient hearing changes, or irritability. The following measures will be taken to prevent these events: (a) patients will be enrolled in a detailed family search and review the medical history to confirm whether the patient has epilepsy, and if it occurs, exclude the patient from the group; (b) an adequate communication will be conducted by the investigator before the intervention to ensure that the patient is in a good position; (c) during the intervention, using Earplugs to minimize hearing damage; (d) the investigator will be closely aware of the patient's status and keep a record; (e) if the patient feels any discomfort, the intervention will be stopped immediately. Subjects may experience irritability and discomfort during MRI scanning. Methods to prevent these adverse events are as follows: check whether there is metal in the patient's body before enrollment, use anti-dry headphones to reduce sound stimulation during the examination; family members can accompany the examination if necessary. If irritation occurs, stop the scan immediately.

### Data Collection and Management

The trial process will be recorded via audio or in a written form to assure the authenticity of the intervention. A case report form (CRF) will be used to collect data. Two data managers will enter the data from the CRF into computer databases and will cross-check the uniformity of the hardcopy and electronic data. During the enrollment phase, each participant will remain anonymous through an assigned identifier. Only the CRF will record the patient's identifying information. All data are confidential to anyone external to the study with the exception of the ethics committee. Experimental data will be used to write clinical research articles. Experimental data will be used to write clinical research articles. Regarding the MRI data, we will obtain consent from the participants to keep the data in the hospital for future studies. During the process of the study, if participants discontinue or deviate from the intervention protocol, we will collect as much data as possible for further analysis.

We will use the following methods to facilitate participants' completion of the trial and follow-up: (a) enhancing communication between investigators and patients to gain patients' cooperation to the extent possible; (b) providing financial compensation according to the number of patients' study visits in order to encourage patients to continue to participate in the trial; and (c) providing free relevant examination results to study patients, including the results of cognitive assessments.

### Statistical Analysis

Baseline comparisons were used to examine potential differences between real-rTMS, sham-rTMS, and cognitive training, which ensured that there are no differences between the three groups. Potential baseline differences between the three groups will be detected. Gender, education, and pathological characteristics (axonal injury or cerebral hemorrhage) will be measured using Chi-square tests. The One-way ANOVA is conducted to analyze the outcome and information of the pre-intervention assessment. The Kolmogorov-Smirnov test will be used to evaluate the normality of distributions. If a normal distribution is confirmed, data will be presented as means and standard deviations (SD) and ANOVA will be used to analyze the effects at T1, T2, T3, and T4, with Bonferroni correction for multiple comparisons as a *post hoc* test. if it does not conform to a normal distribution, it will be tested using Kruskal-Wallis (non-parametric test). *P*-values <0.05 will be considered statistically significant. Data will be analyzed using the Statistical Package for Social Sciences (SPSS) software (v.22.0; SPSS, Inc., Chicago, IL, USA). MRI data will be examined in statistical parametric mapping using independent Student's *t*-tests. The statistical significance is P <0.001. To find other correlated factors that may have an impact on the efficacy of the intervention, correlations between changes in MRI data and changes in behavioral data will be calculated. Only patients completing the rTMS protocol will be included in analyses.

## Discussion

Thus, far, few studies have reported on the effects of rTMS on cognitive improvement in individuals with TBI. We design a random and comparison clinical trial to observe the effectiveness of rTMS and sham rTMS and cognitive training for cognition of TBI patients.

One of the factors to consider when implementing rTMS is the specificity of its stimulation location. the neurobehavioral effects of rTMS can vary dramatically depending on the specific brain region being stimulated. A critical anatomical region for cognitive enhancement through rTMS is the DLPFC. The DLPFC is a brain region involved in cognitive decision-making that regulates task preparation and maintenance as well as task-related attentional focus (33). Numerous studies have indicated that high-frequency rTMS delivered to the left DLPFC may affect attentional control (18, 34). Some studies have also reported on the role of rTMS delivered to the right frontoparietal lobe in the orientation and maintenance of visuospatial attention (20, 35, 36). When low-frequency rTMS was applied to the frontal cortices of healthy volunteers, glucose metabolism was increased at the site of stimulation and in regions of distance connectivity. A recent report by Neville et al. (18) showed no differences in performance on part B of the Connectedness Test (TMT) after 10 sessions of real and sham rTMS in patients with chronic diffuse axonal injury (DAI), indicating that cognitive function was not improved in patients with DAI. However, Tammy et al. (19) reported that 1 Hz of rTMS delivered to the right DLPFC improved cognitive function in TBI patients after 10 sessions. The difference between the previous study is that rTMS will be delivered to different sites including left or right DLPFC.

In our study, we will apply rTMS on bilateral DLPFC, which differs from the previous intervention protocols. It has likewise been reported that rTMS applied to both the left and right DLPFC can be administered in the effective treatment of depression and can improve serum neuron-specific enolase (NSE) and brain-derived neurotrophic factor (BDNF) levels as well as temporal perception in neurologic patients, causing an overall protective effect on neurological function (34, 37, 38). It has been pointed out that HF-rTMS can significantly improve executive function in patients with cerebrovascular disease, and LF-rTMS can improve attention deficit or inattention in patients with depression and schizophrenia (39). In addition to executive function and memory loss, depressed or dryness are also common symptoms in TBI patients, and these long-term mood disorders can also indirectly contribute to cognitive behavior (1). We performed bilateral rTMS on subjects simultaneously, which may improve both the cognitive difficulties driven by neurological injury and the individual's mental status, thus effectively improving the cognitive abilities of TBI patients.

In the proposed study, resting-state functional magnetic resonance imaging (rs-fMRI) will be used to explore the pathophysiological processes mediating TBI cognitive dysfunction through DLPFC stimulation delivered through rTMS. Disruption of brain network connectivity is an important mechanism leading to cognitive dysfunction in TBI (40); cognitive dysfunction may be primarily associated with disruptions to the default mode network (DMN) (41, 42). The DMN is a task-negative network that is preferentially activated when the individual is not concerned with the external environment and involves several internal cognitions, such as autobiographical memory extraction, looking into the future, envisioning others' perspectives, and processing emotional cognition (43). A recent study found that the DMN is not a single network but rather consists of several intertwined networks. The DMN exhibits positive correlations across internal regions as well as negative correlations with relevant regions of external attentional processing (30). A review of 17 original studies conducted in patients with TBI showed that 53% of these studies reported that rTMS promotes increased brain connectivity, 18% reported that rTMS is associated with decreased functional connectivity, and 29% reported no changes in brain connectivity following rTMS (31). The DLPFC is an important component of the DMN network and its use as a rTMS stimulation site may modulate functional connectivity in the DMN. It is possible that connections within and outside the DMN are transmitted through the paracentral hippocampal cortex and posterior cingulate gyrus as nodes for information transmission (44, 45). As shown by Shang et al. rTMS on the left DLPFC alters the resting-state functional connectivity from the DLPFC to the hippocampal parietal, posterior cingulate cortex (PCC)/precuneus regions in the DMN network (16).

As age, education level, TBI severity, and patients' state of consciousness have been reported as factors influencing patients' cognition (8), this study was proposed to conduct subgroup analyses following measurements of baseline levels of RLAS and MoCA to reduce the effects of raw scores on the obtained results. The rTMS in this study applied to two sites of targeting and may have different efficacy in different subjects, providing a new direction for rTMS intervention in cognition in TBI.

## Ethics Statement

The studies involving human participants were reviewed and approved by the Ethics Committee of Nanchong Central Hospital. The patients/participants provided their written informed consent to participate in this study. Written informed consent was obtained from the individual(s) for the publication of any potentially identifiable images or data included in this article.

## Author Contributions

HZ and YQ conceptualized and wrote the study. YZ, HR, YH, ZC, HL, and YP prepared the manuscript and contributed to the study design. YQ reviewed and approved the manuscript for final submission. All authors contributed to the writing of the article and approved the submitted version.

## Funding

This study was supported by the Study and Application of Active Rehabilitation Technology for Cerebrovascular Disease based on the Brain-Computer Interface (BCI) (National Key R&D Plan 2017YFC1308504, 2017YFC1308500). The Sichuan Provincial Medical Youth Innovation Research Project (Q20036).

## Conflict of Interest

The authors declare that the research was conducted in the absence of any commercial or financial relationships that could be construed as a potential conflict of interest.

## Publisher's Note

All claims expressed in this article are solely those of the authors and do not necessarily represent those of their affiliated organizations, or those of the publisher, the editors and the reviewers. Any product that may be evaluated in this article, or claim that may be made by its manufacturer, is not guaranteed or endorsed by the publisher.

## Abbreviations

RLAS: Rancho Los Amigos Scale
DST: Digit Span Test
MoCA: The Montreal Cognitive Assessment
rTMS: repetitive transcranial magnetic stimulation
STT: The Shape Trail Test
HVLT: Hopkins Verbal Learning Test
BVMT: Brief Visuospatial Memory Test
fMRI: functional Magnetic Resonance Imaging.
